# In vitro models of cancer stem cells and clinical applications

**DOI:** 10.1186/s12885-016-2774-3

**Published:** 2016-09-30

**Authors:** Sara S. Franco, Karolina Szczesna, Maria S. Iliou, Mohammed Al-Qahtani, Ali Mobasheri, Julianna Kobolák, András Dinnyés

**Affiliations:** 1 Szent István University, Gödöllö, Hungary; 2 Biotalentum Ltd., Gödöllö, Hungary; 3 Beth Israel Deaconess Medical Center, Department of Medicine, Harvard Medical School, Boston, MA USA; 4 Center of Excellence in Genomic Medicine Research (CEGMR), King AbdulAziz University, Jeddah, Kingdom of Saudi Arabia; 5 Department of Veterinary Preclinical Sciences, School of Veterinary Medicine, Faculty of Health and Medical Sciences, University of Surrey, Guildford, Surrey UK; 6 Department of Farm Animal Health, Faculty of Veterinary Medicine, Utrecht University, Utrecht, The Netherlands

**Keywords:** Cancer stem cells, Cancer, In vitro models, Cancer therapy

## Abstract

Cancer cells, stem cells and cancer stem cells have for a long time played a significant role in the biomedical sciences. Though cancer therapy is more effective than it was a few years ago, the truth is that still none of the current non-surgical treatments can cure cancer effectively. The reason could be due to the subpopulation called “cancer stem cells” (CSCs), being defined as those cells within a tumour that have properties of stem cells: self-renewal and the ability for differentiation into multiple cell types that occur in tumours.

The phenomenon of CSCs is based on their resistance to many of the current cancer therapies, which results in tumour relapse. Although further investigation regarding CSCs is still needed, there is already evidence that these cells may play an important role in the prognosis of cancer, progression and therapeutic strategy. Therefore, long-term patient survival may depend on the elimination of CSCs. Consequently, isolation of pure CSC populations or reprogramming of cancer cells into CSCs, from cancer cell lines or primary tumours, would be a useful tool to gain an in-depth knowledge about heterogeneity and plasticity of CSC phenotypes and therefore carcinogenesis. Herein, we will discuss current CSC models, methods used to characterize CSCs, candidate markers, characteristic signalling pathways and clinical applications of CSCs. Some examples of CSC-specific treatments that are currently in early clinical phases will also be presented in this review.

## Background

Despite years of intensive experimental efforts and development of several novel treatment strategies, cancer is still a leading cause of mortality. New knowledge of the fundamental mechanisms underlying initiation of carcinogenesis can lead to a dramatic shift in the field of cancer research and treatment. This innovative understanding combines cancer and stem cell biology. The hypothesis that cancer is caused by the expansion of a population of cells with stem cell characteristics has attracted considerable attention [1–3]. The model suggests a hierarchical organization of tumour cells, with self-renewal and differentiation abilities, that are capable of regenerating a new tumour in vivo with the same heterogeneity as the primary tumour [1]. This type of cells, identified as CSCs or tumour-initiating cells, possess unrestrained proliferative abilities, resistance to apoptotic cues, and aptitude to establish tumours in immunodeficient mice [4, 5]. The cellular hierarchy was first identified in hematopoietic malignancies such as myelogenous leukaemia [6, 7], and later on in solid tumours [4], based on the expression of specific cell surface molecules.

CSCs have been reported to be resistant to conventional chemo- and radio-therapy [8, 9]; therefore, novel therapies integrating the current oncologic treatments should be identified and developed. The existing therapies generally target highly replicating cancer cells that constitute the bulk of the tumour but may not eradicate CSCs. For this reason, instead of trying to eliminate the bulk of the tumour or reduce its size, development of therapies aiming to eradicate the resistant CSCs would be a more efficient and rational approach [10]. Furthermore, it is important to identify and characterize CSCs in order to improve our knowledge about the biology of the tumour and carcinogenesis.

Identification of CSCs is a complex process and it relies on different strategies: expression of specific surface markers, in vitro assays such as anchorage-independent growth assays (tumour sphere assays) and in vivo limiting dilution assays, among others [11]. In this review, we will summarize the most recent cell-based in vitro models of CSCs and the promising clinical applications derived from these models, from biomarker discovery and microenvironment analysis to the identification of novel oncologic targets and therapies.

## History and current view of the CSC concept

The CSC model suggests that a subset of cancer cells displaying self-renewal and pluripotency properties is responsible for the regeneration of malignant tissues and their homeostasis [10].

**Fig. 1:**
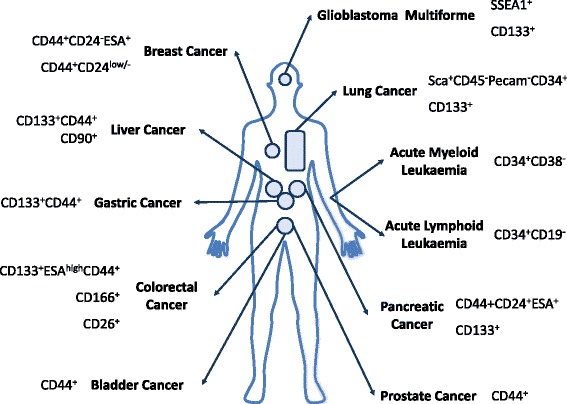
Most common cell surface markers currently used to identify CSC subpopulations from different types of cancer

In 1875 Cohnheim proposed the first CSC hypothesis, also called the “embryonal-rest theory”, that suggested the presence of embryonic-like cancerous cell remnants in adult tissues which develop into cancer in a non–spontaneous way [12]. In 1994 Dick and colleagues demonstrated that leukaemia initiating stem cells (LSCs), present in the blood of leukaemia patients, may induce acute myelogenous leukaemia (AML) when transplanted into severe combined immunodeficient (SCID) mice [6]. This was the first experimental evidence in support of the CSC theory, demonstrating the existence of cancer stem-like cells. A broad spectrum of cell surface stem cell markers (e.g. CD133, CD44, and CD24) allowed the identification of CSCs in human solid tumours, including brain, breast, prostate, pancreas, liver, ovary, skin and colon cancers, and melanoma [13–20] (Fig. 1).

Recently, two models that illustrate the organization of CSCs within the tumour have been proposed. According to the “stochastic model” (or “clonal evolution model”), initially described by Nowell and colleagues, every cancer cell in the tumour could gain the ability to self-renew and differentiate to the numerous and heterogeneous lineages of cancer cells that compromise the tumour. In this way, they were capable of repopulating the entire tumour [21]. On the other hand, the “hierarchy model” is based on the assumption that every tumour is a heterogeneous conglomerate of cancer cells and only a minority of them possess CSC properties [22].

During the past years, the origin of CSCs has been the centre of investigation, and in most cases it remains unknown and controversial, as shown by different studies on LSCs [23, 24]. The identification of CSCs origin is very complex as the process of malignant transformation into CSCs may occur in different stages and within a very limited subpopulation of cells, therefore difficult to reach and study in detail. Nonetheless, the literature suggests adult stem and/or progenitor cells as the potential cell of origin of CSCs [10]. Adult stem cells are long lived cellular reservoirs with the mission to replenish damaged/senescent cells contributing to tissue homeostasis. According to Feinberg’s “epigenetic progenitor model of human cancer” these cells live long enough to accumulate genetic and epigenetic changes that help them rearrange their microenvironment in such a way so that they can promote the origin of CSCs [25].

Normal adult stem cells have an ability to immortalize themselves via self-renewal and to generate mature cells of appropriate tissue through differentiation. Biology of stem cells is a relevant source of knowledge for cancer studies because of the similarities in the mechanisms that regulate self-renewal of normal (adult) stem and cancer cells [26]. Indeed, normal adult stem cells and CSCs share many characteristics. In addition to i) self-renewal and differentiation properties via asymmetric division, ii) CSCs and adult stem cells rely on the same signalling pathways; like Wnt, Hedgehog, Notch, among others, to control self-renewal, iii) they have similar metabolism (showing preference to oxidative glycolysis), iv) they express ATP-binding cassette (ABC) efflux transporters that protect cells from toxic compounds, v) express similar surface markers such as CD133 and CD44, vi) have enhanced anti-apoptotic, DNA-repair and oxidative stress-protective mechanisms, vii) exhibit a slow-cycling phenotype related to a quiescent state, viii) have similar genetic and epigenetic profiles, ix) have extended telomeres, and x) exhibit long life spans [27, 28].

In order to comprehend the connection between CSCs and normal stem cells, the transcriptional program in intestinal stem cells and colorectal carcinoma [29], as well as lung stem cells and lung CSCs [30], were studied. Interestingly, both studies revealed a significant overlap in the gene expression profiles between the two types of cell populations, mainly associated with self-renewal, angiogenesis, migration, and anti-apoptosis. Moreover, some proteins highly expressed in embryonic stem cells (ESCs), such as PRC2-regulated genes and the transcription factors Klf4, Nanog, Oct4, and Sox2, are frequently present in poorly differentiated tumours with adverse clinical outcome [31, 32]. The importance of Nanog and Oct4 in cancer research is supported by the elevated expression of these transcription factors in lung and ovarian cancer cells, which results in drug resistance and promotion of the epithelial-mesenchymal transition (EMT) [32, 33]. Moreover, some groups suggest that CSCs could also originate from more committed progenitors that acquire stemness-related features as an effect of accumulation of epigenetic and/or genetic changes [34, 35].

Metastasis, and not the primary tumour itself, is usually the main cause of death in cancer patients. Metastasis demands that cancer cells detach from the primary tumour site, travel systemically and form new tumours elsewhere in the body. Metastasis in cancer might be associated with the existence of a subpopulation of CSCs which initiate the formation of secondary tumours in distant organs and promote their proliferation, followed by the recruitment of new vasculature, or angiogenesis [36]. Although the current knowledge about the association of CSCs with metastasis is low, recent evidence supports the idea that EMT markers, known to be implicated in tumour cell migration, are expressed in CSCs [37]. These cells might be the precursors of the metastatic CSCs. Cancer cells might lose their epithelial characteristics by lack of polarity or intercellular adhesion, and gain mesenchymal properties followed by migration to different parts of the body [38]. In 2004 Yang and colleagues proposed EMT as being a required step in breast cancer metastasis [39]. In Scheel et al. (2011), the authors suggest that any given normal or neoplastic epithelial cell type utilizes the same network of paracrine and autocrine signals from the microenvironment in order to reach a certain EMT potential and maintain itself in a mesenchymal stem cell state with enhanced metastastic properties. In transplantation assays this is manifested by an increase of mammosphere forming cells with CD44^+^CD24^-^ phenotype [40]. Many different genetic and epigenetic modifications contributing to adult stem and progenitor cell homeostasis can subsequently reprogram these cells to acquire some features that in conjunction with a potential “tumour promoting environment”, may lead to cancer cells and CSC formation [3].

Proper elimination of CSCs might avoid cancer recurrence, and therefore, it is important for a successful cancer therapy. Indeed, CSCs are resistant to anti-tumour therapies, which preferentially eliminate cells with more differentiated phenotypes. This results in surviving CSCs that are responsible for tumour recurrence [41, 42]. The most important evidence supporting the critical role of CSCs in tumour regeneration has emerged from breast cancer studies. In this type of cancer up to 25 % of recurrences may appear after 10 years of the first successful therapy, and these cancers still have the properties similar to the primary tumours [43, 44]. This observation can be related with the presence of a small population of surviving CSCs more resistant to anti-tumour treatments than non–stem cancer cells that may be responsible for tumour relapse after initial successful therapy [9]. In a more recent study it was described that breast cancer metastasizes in bone marrow, where it acquires characteristics of slow cycling cells with enhanced chemoresistance and gap junctional intercellular communication (GJIC) common with the stroma [45].

## Cell-based models of CSCs

### Approaches to isolate the cancer stem-like cell population

Detection and quantification of CSCs can be carried out through in vivo or in vitro assays. The introduction of cells either from human primary tumours or cancer cell lines into immunodeficient mice, like non-obese diabetic severe combined immunodeficient (NOD/SCID) mice, offers a sensitive system to identify CSCs. Although this system is commonly used, it has some weaknesses, such as short life span of the mouse models, inadequacy of the CSC-specific cytokines and NK-driven residual innate immunity and failure to represent the tumour or disease microenvironment [46].

Quintana was first able to show that he can dramatically increase the sensitivity of the assay in recognizing CSCs by employing a “modified xenotransplantation assay”, such as the NOD scid gamma (NSG) mice, which lack the interleukin-2 receptor gamma chain [47]. With this model, almost 25 % of melanoma cells from patients were able to form new tumours in vivo. This implies that CSCs may not be as rare as previously estimated, highlighting at the same time the vital role of microenvironment in supporting tumour growth. Using this approach, Ishizawa et al. (2010) determined that the frequency of CSCs in many tumours is up to 10-fold higher in NSG mice, but generally it remained low (less than 0.04 %) overall [48]. Similarly, Kuperwasser and colleagues employed genetically modified mouse models for breast cancer growth and metastasis, showing that species-specific stromal-niche interactions are critical for the in vivo growth and osteotropic metastasis of CSCs [49]. More “humanized” murine models should be employed in the future, which will be more permissive of implanted human cells to reconstitute their natural microenvironment [50].

By being a rare population within the tumour tissue, CSCs are difficult to isolate and study, which limits our ability to monitor the development and the pathogenesis of the cancer. This issue makes it necessary to develop cell-based models of cancer stem-like cells. Many in vitro assays for detection and identification of CSCs have been used, though a non-universal method can be applied [51]. The application of these CSC in vitro models has many benefits as it would permit the increment of our knowledge about CSCs, together with tumour biology, microenvironment, carcinogenesis, biomarker discovery, and could lead to improvement and progress of oncologic therapies, among other applications.

Recently published literature has employed different in vitro approaches to reach the CSC population. According to these publications, CSCs can be mainly obtained from cancer cell lines or primary tumours through i) reprogramming [32, 52–69], ii) the expression of specific surface markers [15, 70, 71], iii) detection of the side population [72–78], iv) selection of cells resistant to anoikis [79], v) or based on the application of specific culture conditions, among other techniques [80–83].

### Reprogramming of cancer cells as a way to model cancer

Current cancer models are not sufficient to tackle the heterogeneity of human cancers. Animal models of cancer have been used for decades however, distinctions are observed between animal and human cancers. Cancer cell lines, although inexpensive and immortal, do not represent the primary tumour and disease progression, and they cannot easily be replaced by the primary cells isolated from tumours, that are limited and complex to maintain. In this way, in vitro models of carcinogenesis derived from reprogramming patients’ cancer cells offer promise for the deciphering of the early mechanisms of the development and progression of cancers from pluripotent cells [84].

Historically, reprogramming back to pluripotency involved either blastocyst injection [85], or nuclear transfer (NT) [86], or lately, the exogenous forced expression of specific sets of transcription factors, known as “induced pluripotent stem cell (iPSC) technology” [32, 52–69] (see Table 1 for more details).

**Table 1:** Summary of in vitro cancer stem cell models based on cancer cell reprogramming

Type of cancer		Species	Cancer cell line/tumour	Reprogramming factors	Characterization of the pluripotency state	Teratoma ?	Chimera ?	Tumour description	Ref.
Gastro intestinal cancer	Pancreatic cancer	Human	Primary solid tumour from patients	Oct4, Sox2, Klf4, c-Myc	Nanog, Oct4, Rex1, Ssea-4, sphere formation and differentiation into three germ layers	Yes	-	PanIN precursors to PDAC, evolution to the invasive stage	[69]
	Liver cancer	Human	Cancer cell line PLC/PRF/5	mir-302	Nanog Oct4 Sox2, Rex1, alkaline phosphatase activity, sphere formation and differentiation into three germ layers	-	-	-	[99]
		Human		Oct4, Sox2, Klf4, c-Myc	Nanog, Oct4, Klf4, Rex1, Ssea-4, Tra-1-60, sphere formation and differentiation into three germ layers	-	-	-	[56]
				Oct4, Sox2, Klf4, c-Myc	c-Myc, Sox2, Oct4, Klf4, Nanog, Rex1, Tra-1-60, Tra-1-81, Tra-2-49, Ssea-4, multi-differentiation potential into three germ layers	-	-	-	[60]
	Cholangio carcinoma	Human	Cancer cell line HuCC-T1				-	Enhanced tumorigenicity	
	Colorectal cancer	Human	Cancer cell line DLD-1			-	-	-	
				Oct4, Sox2, Klf4, c-Myc	Nanog, Oct3/4, Sox2, Klf4, c-Myc, Rex1, Ssea-4, Tra-1-60, Tra-1-81, Tra-2-49, sphere formation and differentiation into three germ layers	-	-	-	[57]
		Human	Cancer cell lines SW480 and SW620	Oct4, Sox2, Klf4, c-Myc	Sox2, Oct4, Lin28, sphere formation	-	-	Enhanced tumorigenicity	[59]
		Human	Cancer cell line SW480	Oct4, Sox2, Klf4	Sphere formation	-	-	Enhanced tumorigenicity	[98]
Leukaemia		Human	Cancer cell line KBM7	Oct4, Sox2, Klf4, c-Myc	Oct4, Sox2, Rex1, FGF4, TDGF1, Nanog, GDF3, Lin28, Zic3, CD9, Tra-1-81, alkaline phosphatase activity, sphere formation	Yes	-	-	[66]
		Human	CD34^+^ cells from patients with CML	Oct4, Sox2, Klf4, c-Myc	Oct4, Sox2, Klf4, Nanog, Lin28, Rex1, Ssea-4, Tra-1-60	Yes	-	-	[67]
		Human	Bone marrow mononuclear cells from patient with CML	Oct4, Sox2, Klf4, c-Myc, Nanog, Lin28,	Oct4, Sox2, Nanog, Lin28, GDF3, Rex1, GDF3, Ssea-3, Ssea-4, TrA-1-60, Tra-1-81, alkaline phosphatase activity	Yes	-	-	[68]
Lung cancer		Human	Cancer cell lines H358 and H460	Oct4, Sox2, Klf4, c-Myc	Nanog, Sox2, FGF4, Oct4, Tra-1-60, alkaline phosphatase activity, telomerase activity, sphere formation and differentiation into three germ layers	-	-	-	[54]
			Cancer cell line A549	Oct4, Sox2, Nanog, Lin28, HIF	Alkaline phosphatase activity (partial reprogramming)	-	-	Highly aggressive malignant solid tumors, high mitotic index, local invansiveness	[55]
				Oct4, Nanog	Oct4, Nanog, sphere formation	-	-	Enhanced tumorigenicity and metastasis	[32]
		Murine	Lewis lung mouse carcinoma cell line LLC	Conditioned medium for carcinoma cells (role of microenvironment)	Oct3, Oct4, Nanog, Rex1, Ras, Esg1, Cripto	Yes	-	Spheroid and tumor formation in mice, high mitotic index, angiogenesis in vivo	[83]
Sarco mas	Osteosarcoma	Human	Cancer cell lines SAOS2, HOS, MG63	Oct4, Nanog, Sox2, Lin28, Klf4 and c-Myc	Oct4, Nanog, Sox2, Lin28, Klf4, c-Myc, Tra-1-81, Ssea-4, Rex, alkaline phosphatase activity	-	-	-	[61]
	Liposarcoma	Human	Cancer cell line SW872						
	Ewing’s sarcoma	Human	Cancer cell line SKNEP						
		Human	Cancer cell line CHLA-10	Oct4, Sox2, Klf4, Nanog	Tra1-160, Ssea-3, Ssea-4, alkaline phosphatase activity	Yes	-	Various levels of undifferentiated cells, embryoid body and tumor formation in mice	[110]
Breast Cancer		Human	Cancer cell line MCF-7	Oct4, Sox2, Klf4, c-Myc	Sox2, Ssea-1, Ssea-4, Tra-1–60, Tra-1–81, alkaline phosphatase activity, sphere formation		-	Enhanced tumorigenicity, highly aggressive tumors	[63]
				Sox2	Ssea-4	-	-	Enhanced tumorigenicity	[64]
		Murine	Mammary carcinoma cell line MCE12	-	-	-	-	-	[83]
Prostate cancer		Human	Prostate cancer-associated stromal cells from primary tumours	Oct4, Nanog, Sox2, Lin28	Oct4, Nanog, Lin28, Sox2, alkaline phosphatase activity	-	-	-	[65]
		Human	Cancer cell line PC3	mir-302	Oct4, Nanog, Sox2, Ssea-3, Ssea-4, sphere formation and differentiation into neuronal cells	Yes	-	-	[53]
Melanoma		Murine	Cancer cell line Colo	mir-302	Sphere formation and differentiation into neuronal cells		-	-	[53]
			Cancer cell line R545	Oct4, Klf4, c-Myc	-	Yes	Yes	-	[52]
			Melanoma cell line B16	-	-	-	-	-	[83]

Similarly to normal somatic cells, human cancer cells have of late been successfully reprogrammed into a pluripotent state, as shown for gastrointestinal [57–60, 69], breast [62–64, 87], prostate [65], bone [61], liver [56], and lung [32, 54, 55] cancers, melanoma [52, 53, 88], and leukaemia [66–68]. By introducing nuclei from mouse melanoma cells into enucleated oocytes, Hochedlinger and colleagues (2004) established ESC lines from blastocysts, proving that cancer cells can be reprogrammed into a stem state disregarding the genetic aberrations. These ESCs had the potential to generate teratomas and to form chimeras that develop high penetrance melanomas [88]. Similarly, Blellock and colleagues were able to generate NT-derived ES cell lines from human embryonal carcinoma (EC) cells, with developmental potential similar to the respective donor EC cells [89].

In 2006 Yamanaka and Takahashi demonstrated that additionally to NT or blastocyst injection, nuclear reprogramming can be achieved by applying iPSC technology. Yamanaka’s group established a panel of specific transcription factors, Oct4, Sox2, Klf4, and c-Myc (OSKM, or Yamanaka factors), which were previously shown to be oncogenic, with the ability to induce an ESC-specific gene expression module into somatic cells, converting them into pluripotent cells, the iPSCs [90]. This technology has been updated and applied effectively to somatic cells of many species, such as mouse [90], rat [91], rabbit [92], pig [93], rhesus monkey [94], and human [95–97], resulting in the generation of pluripotent ES-like cells with the capability to differentiate into cells of the three germ layers [90]. Similarly to normal somatic cells, malignant cells either from cancer cell lines [32, 52–55, 57–64, 66, 98, 99] or primary tumours [65, 67–69] from several cancer types have been successfully converted to iPS-like cells, or induced pluripotent cancer cells (iPCs) [55, 57, 66, 69] (Table 1).

In the last half-decade many research groups employed iPSC technology in their research involving human cancer cells, mainly through the overexpression of Oct4, Sox2, Klf4, and c-Myc factors [54, 57, 66, 69] (summarized in Table 1). The obtained reprogrammed cells are pluripotent as defined by all in vitro and in vivo criteria used to define pluripotent stem cells generated from somatic cells [90, 95], i.e. they express ESC markers, telomerase activity, have self-renewal abilities and are pluripotent by generating cells of all three germ layers; as shown by the formation of spheres in vitro, and teratomas in immunocompromised mice. When Oct4, Klf4, and c-Myc were overexpressed in a murine cancer cell line of melanoma, the generated iPCs were able to form viable chimeric mice after injection into blastocysts [52].

Although Yamanaka’s set of iPSC factors are the most commonly used in research, the replacement of c-Myc and Klf4 with Nanog and Lin28 was implemented to human fibroblasts by Thomson’s group, as c-Myc may cause death and differentiation of ESCs [95]. This new group of transcription factors, together with the overexpression of hypoxia-inducible factor (HIF) could convert lung cancer cells into partially reprogrammed cells capable of generating highly aggressive malignant tumours when injected into immunocompromised mice [55]. Alternatively, other groups have demonstrated that not all four transcription factors are required to reprogram cancer cells. This conclusion came from analysis of primary tumour specimens from patients. Chiou et al. (2010) showed that CD133^+^ lung CSCs co-express Oct4 and Nanog when compared to CD133^-^ cancer cells. When overexpressed in A549 cancer cells, Oct4 and Nanog induced the formation of cancer stem-like cells and enhanced tumorigenesis and metastasis in mice [32]. A similar approach was used in breast cancer where early stages of carcinogenesis were linked with Sox2 expression. When overexpressed alone in a breast cancer cell line, Sox2 stimulated the formation of spheres in vitro and tumour in vivo, demonstrating its function in the pluripotency preservation [62]. In other studies, Sox2 was dispensable in reprogramming of melanoma cells into iPSCs [52].

Alternative methods of reprogramming, where no integration in the genome occurs, have been published. In a study proposed by Ying and co-workers (2008), the family of microRNA (miRNA) miR-302, highly expressed in ESCs, was transfected into cancer cells generating ES-like pluripotent cells with self-renewal and multipotential differentiation properties [53]. A similar technology was successfully applied to hepatocellular carcinoma cells as shown by the group of Mori in 2014 [99]. On the other hand, reprogramming of cancer cells can be achieved through the application of small chemical molecules. When breast cancer cells were treated with valproic acid (VA), aldehyde dehydrogenase (ALDH)-positive cells with a superior efficiency of mammosphere formation and tumorigenic ability were generated [100].

Though the iPSC process has been applied successfully to cancer cells from both primary tumours and cancer cell lines, it is time-consuming and less efficient compared to the reprogramming of non-tumorigenic somatic cells [57, 63, 67, 69, 101]. This observation may be due to the availability of certain number of cancer cells that contain a particular genetic and epigenetic status like aneuploidy or mutations in components involved in the epigenome [102]. Albeit a lower reprogramming efficiency has been described for cancer cells, some exceptions have been observed. Zhang et al. (2013) observed a slight augmentation of efficiency, probably associated with loss of pre-existing tumour suppressors [61]. As shown for normal somatic cells, the improvement of the reprogramming efficiency can be achieved through the overexpression of mir-302 [53] or HIF [55], deficiency of p53 [58] or Ink4/Arf [103], hypoxia [55, 58], or through the treatment with histone deacetylase (HDAC) inhibitors [e.g. azacitidine (5-azaC)] [104], VA [100], suberoylanilide hydroxamic acid (SAHA), or trichostatin A (TSA) [105]. When reprogramming factors were expressed together with HIF in lung cancer cells, the generated iPCs had enhanced tumorigenesis, showing the importance of hypoxia in the improvement of the reprogramming efficiency [55]. In 2009 Yamanaka proposed the “stochastic model”, rather than the “elite model”, to explain the low efficiency observed during the reprogramming process of normal somatic cells. Briefly, the “elite model” states the existence of a small population of cells that can be reprogrammed partially or completely. Alternately, the “stochastic model” states that most cells may undergo the process of reprogramming but just a minority completes it [106]. Lai and colleagues (2013) assessed this issue in reprogrammed cancer cells and concluded that the reprogramming process of these cells may follow the “elite model” [107]. According to the published results, only a small subpopulation of cells was selected for reprogramming as all obtained iPCs were free of mutations, unlike the parental cells. The contradictory results between iPSCs and iPCs may be related with the differences observed between normal somatic cells and cancer cells.

iPSCs tend to differentiate into the same tissue lineage from which they originate. Erasure of the epigenetic memory (methyl-DNA-marks) during factor-based reprogramming seems to be inefficient, leading to the maintenance of a “residual epigenetic memory” within the iPSCs [108]. Kim and colleagues performed a high-throughput array-based analysis of the methylome of iPS cells from different sources, in which they observed DNA methylation signatures reminiscent of the tissue of origin. Epigenetic memory may explain why blood-derived iPSCs differentiate with higher efficiency into blood when compared to fibroblast-derived iPSCs [108]. A similar observation was demonstrated for iPCs, which tend to generate the cancer type from which they are derived [69]. This can be due to a partial reprogramming of cancer cells. Although the reprogramming of cancer cells can generate cells with similar characteristics to ESCs, as shown by the transcriptome analysis undertaken by Mahalingam and co-workers [54], iPCs may not be so similar to ESCs as it was thought. In 2013 the group of Matushansky published an interesting work where the degree of differentiation reversion was evaluated after reprogramming sarcoma cells [61]. When the level of differentiation was compared, the gene expression and DNA methylation analysis denoted that reprogrammed sarcomas are closer to sarcomas and to partially reprogrammed fibroblasts than to fully reprogrammed fibroblasts and ESCs [61]. Similar conclusions were drawn by Menendez and colleagues (2013), where reprogrammed MCF-7 cells were in a status between differentiated cancer cells and iPSCs [63]. Taken together, these suggest that reprogrammed cancer cells seem to reach a “minimum state of differentiation” which is enough to confer them a pluri-differentiation potential. Alternatively, as suggested by Nagata et al. (2012), a pluripotent state may be achieved but not necessarily an ESC state [81].

Some groups have shown that reprogramming of cancer cells into a pluripotency state can decrease the tumorigenic capability of reprogrammed cells compared to parental cells [52, 54, 57]. This observation can be associated with the epigenetic reset that can drive the downregulation of the mechanisms (i.e. silencing of oncogenes) related with the tumorigenic behaviour of the cancer cells [54, 102]. In fact, a study by Wang and co-workers (2012) demonstrated that reprogrammed lung cancer cells hold a rectified epigenetic profile that may result in a reduction of the tumorigenic potential of these cells. The downregulation of genes usually expressed in non-small cell lung cancer (NSCLC) (e.g. KRT19 and *S100P*) might be associated with DNA methylation occurred during reprogramming, indicating the importance of DNA methylation to tumorigenesis [54]. This characteristic has become of interest for drug development and therapy. Moreover, when reprogrammed cancer cells are induced to differentiate into a germ layer diverse of the tissue from which they are derived, these cells lose the tumorigenic ability in vivo [61, 109]. Hence, when glioblastoma-initiating cells were induced to differentiate into a mesoderm lineage, no malignant brain tumours developed in mice [109]. An analogous observation was made by Zhang et al. (2013). When reprogrammed sarcomas were terminally differentiated either into their own or into an alternate lineage of origin and inoculated in NSG mice, in both cases no tumours were detected [61].

**Fig. 2:**
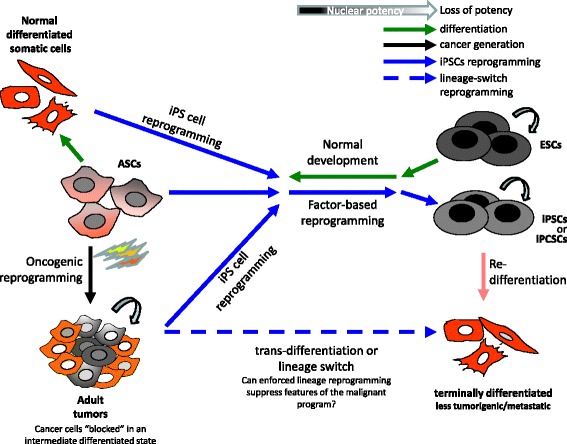
Cancer stem cells reprogramming as an emerging tool in modelling cancer. The normal development (*green arrows*) denotes a passage from a pluripotent (zygote) to a “less potent state” (terminal differentiated adult tissues). During this process, adult stem cells (ASCs) in adult tissues suffer multiple tumorigenic “hits” that lead to the generation of “aberrantly reprogrammed” cancer cells, forced to be maintained in an intermediate degree of cellular differentiation (*black arrow*). Induced pluripotency is now being employed on cancer cell lines or patients’ tumours (named induced pluripotent cancer stem cells or iPCSCs) (*blue arrows*). iPCSCs cells epigenetically and transcriptionally resemble the ESC state and the cancer genome seems to be repressed in pluripotent state. In some cases the iPCSCs may exhibit early stage phenotypes corresponding to partial expression of the reprogrammed cancer genome, constituting in this way a live cell model to study cancer progression [69] (reviewed in [84]). Moreover, these iPCSCs have the ability to re-differentiate (*orange arrow*) back to the original or a different terminal differentiated cell lineage, losing along this process their tumorigenic and metastatic properties [110]. Alternatively, the induction to more differentiated state can occur directly from the adult tumour (in this case “trans-differentiation”-dashed blue arrow), without the need to pass first from the pluripotent state [61]. It is important to clarify the mechanisms controlling these transitions, as the ability to exogenously manipulate the stemness and differentiation of a tumour might hold promise as a therapeutic strategy in the near future

Understanding the transitions among different states and acquiring the ability to exogenously manipulate the stemness and/or differentiation of normal but most importantly of tumours may hold promise as a therapeutic strategy in the near future (Fig. 2). In recent years, induced pluripotent stem cell reprogramming technology offered the possibility of generating clinically valuable pluripotent stem cells (named iPSCs) from fully differentiated somatic cells. The same reprogramming strategy is being now employed on cancer cell lines or patients’ tumours (named induced pluripotent cancer stem cells or iPCSCs) (blue arrows in Fig. 2). iPCSCs cells epigenetically and transcriptionally resemble the ESC state and the cancer genome “seems to be repressed” in the pluripotent state. In some cases the iPCSCs may exhibit early stage phenotypes corresponding to partial expression of the reprogrammed cancer genome, constituting, in this way, a live cell model to study cancer progression [84]. Moreover, these iPCSCs have the ability to re-differentiate (orange arrow in Fig. 2) back to where they originated or other terminal differentiated cell lineage, losing along this process their tumorigenic and metastatic properties [110]. Alternatively, as it was shown in sarcomas, the induction to more differentiated state can occur directly from the adult tumour, in this case named “trans-differentiation” (dashed blue arrow), without the need to pass first from the pluripotent state [61]. In the case of sarcomas, it was shown that various types of sarcomas can be trans-differentiated into multiple mature connective tissues (dashed blue arrow in Fig. 2), abolishing their tumorigenic potential as measured by the ability to further proliferate in vitro (on soft agar) or in vivo (xenograft formation), without the need to be reprogrammed first in the ESC state [61]. Similarly, it was found that the epigenetic switch (mainly tumour suppressor hypo-methylation) in trans-differentiated glioblastomas inhibits their tumorigenicity when injected into mice [102]. Trans-differentiation of cancer cells to terminal differentiation via addition of chemical agents i.e. retinoids for acute promyelocytic leukaemia (APL) [111], peroxisome proliferator activated receptor gamma (PPARg) agonists for liposarcoma [112], or epigenetic drugs [113], could potentially be preferable with respect to clinical application compared to classical factor based reprogramming.

### Other cell-based models of CSCs

Cancer cells with tumorigenic potential can be identified based on their capacity to form tumours when injected into mice. This approach allowed the isolation of the CSC population from breast tumours based on a specific phenotype of these cells, CD44^+^CD24^-/low^ [1]. The idea to identify CSCs by flow cytometry comes from leukaemia where LSCs constitute a population of CD34^+^CD38^-^ cells capable of generating leukaemia in immunosupressed mice [114]. The prospect of CSC identification based on the expression of specific surface markers opened the way to their detection in other solid tumours: brain and lung (CD133^+^), prostate, gastric, and ovarian (CD44^+^) [13–15] cancers [16, 17], among others [18–20]. A more detailed description of the current surface markers used to identify CSCs can be found in Fig. 1 [reviewed by Allegra et al. (2014)] [2]. A unique combination of surface markers has not been identified so far as their universal application to most cancer types and subtypes is limited. Despite the fact that CD44 and CD24 are expressed in most tumours and are commonly used to identify CSCs, they are not specific to all cancers [115], and in addition, they can be also found in ESCs. The identification of surface markers specific to CSCs has allowed their sorting from either cancer cell lines or primary tumours through cytometric-based technologies or immunomagnetic beads [70]. In a recent publication, CD44^+^CD24^−/low^ breast CSCs were isolated from the MCF-7 cell line and evaluated for their resistance to known hormonal therapy. Results demonstrated that the breast CSC population was resistant to doxorubicin but not to tamoxifen [71]. Research undertaken by Takaish and colleagues identified a subpopulation of CD44^+^ cells within gastric cancer cell lines with stem cell properties, such as self-renewal ability and capacity to generate differentiated progeny, and increased resistance to chemo- and radio-therapy. This CD44^+^ population had tumorigenic potential both in vitro and in vivo, unlike CD44^-^ cells [15]. Special care should be applied when evaluating the surface markers by flow cytometry as their integrity might be affected by the dissociation agents. When different digestive agents were compared with regards to their effect on the presence of CD44 and CD24 surface markers in a breast cancer cell line, differences were observed. Indeed, enzymatic digestion affects the identification of CSCs by flow cytometry; trypsin being more aggressive when compared to accutase [116]. Enzyme-free buffers or special tissue engineered surfaces coupled with temperature reduction could be considered as a replacement.

In addition to cell surface markers, the activity of intracellular enzymes, such as ALDH, can be used to enrich CSCs. The stem cell marker ALDH, a detoxifying enzyme highly expressed in stem cells [117], has been commonly used in the identification of CSCs. Charafe-Jauffret and colleagues (2009) evaluated several breast cancer cell lines to conclude that the majority were ALDH^+^ and that this cell population possessed CSC properties [80]. Similar results were obtained for LSCs in AML [118] and malignant breast stem cells in breast cancer [119].

The fluorescent dye exclusion, or side population (SP) phenotype, is another property of stem cells that can be used to identify CSCs. Cells that extrude fluorescent dyes such as Hoechst 33342 are defined SP cells and can be easily identified by the flow cytometry technique. This characteristic is related to the high expression levels of ABC transporters family members, such as ABCG2 transporter. When the SP approach is applied to cancer cells, stem-like cells with tumorigenic abilities and resistance to chemotherapy are obtained [77, 78]. Indeed, ABC drug transporters have been shown to protect CSCs from chemotherapeutic compounds, a characteristic that could be useful for the development of new therapies against these transporters. Huang et al. (2009) identified in human esophageal carcinoma cell lines a subpopulation of cells with CSC properties with higher tumorigenicity, when applying the SP assay [76]. Similar results were obtained in glioma [72], ovarian [73], and pancreatic [74] cell lines. Although SP^+^ cells were showed to be more tumorigenic when compared to SP^-^ cells, ABCG2^+^ and ABCG2^-^ cancer cells were shown to be similar [75].

An effective method to isolate CSCs is based on their resistance to anoikis. Anoikis is a type of programmed cell death, which is activated when cells detach from the surrounding extracellular matrix. This process prevents the growth of adherent-independent cells. The resistance to this mechanism may explain cancer progression and metastasis [120]. For this reason the selection of cells resistant to anoikis may be used as an assay to obtain CSCs. Harrison et al. (2010) reported that anoikis resistant cells from breast cancer cell lines and primary human tumours were mainly ESA^+^CD44^+^CD24^low^ with self-renewal and tumorigenic properties in vitro and in vivo, therefore classified as breast CSCs [79].

Other approaches use specific culture conditions to obtain CSCs such as selection through i) chemotherapy enrichment, ii) growth of stem cells in media derived from cancer cell lines, iii) suspension culture, and iv) repetitive cycles of hypoxia and reoxygenation, among other techniques. Lu and Labhasetwar (2013) established a drug resistant MCF-7 cell line obtained after a long period of treatment with doxorubicin. The resultant cells were described to have CSC characteristics [121]. Tang and colleagues (2011) observed that methotrexate-resistance osteosarcoma cells exhibited SP phenotype, expressed CSC markers and were more tumorigenic when compared to parental cells [122]. When mouse breast cancer cells were treated with pacilitaxel and epirubicin in serum-free condition and then maintained in suspension culture, they became mostly CD44^+^CD24^-^ cells together with higher tumorigenic potential [123]. Instead of long-term drug treatment, the group of Seno (2012) hypothesized that CSCs may be generated when stem cells are subjected to the influence of a tumour microenvironment. Indeed, when mouse iPSCs were maintained in media derived from a lung cancer cell line, cells with CSC-like properties appeared. Cells were described to be tumorigenic when injected in vivo, to possess the ability to self-renewal, and to express stem cell markers [83].

A variety of 3D in vitro sphere forming assays have been developed in order to obtain cancer stem-like cells. These in vitro assays involve mainly sphere formation in low-adherent stem cell culture conditions, a model described as tumourspheres. Often stem-like cell culture conditions include the growth of cells in low-density conditions, to avoid cell aggregation, in media supplemented with epidermal growth factor (EGF), hydrocortisone, insulin, progesterone, and/or heparin in the absence of foetal bovine serum (FBS) [124]. This system has been employed to identify cells with self-renewal and differentiation abilities and therefore to enrich CSCs, as described already for liver [125], pancreatic [126], and oesophageal [127] CSCs. This type of assay displays some disadvantages as it may require specific culture conditions and components, otherwise CSC differentiation or expansion limits may occur. Furthermore, this system does not fully reproduce the tumour from which the cells are derived, especially its structure and/or microenvironment [46]. In addition to the identification of cancer stem-like properties, the tumourspheres were proved to be resistant to chemotherapy and tumorigenic [127]. This technique presents some disadvantages, including the low number of successfully isolated CSCs, spontaneous differentiation, and apoptosis [128, 129]. Louie et al. (2010) developed a system to enrich CSCs without genetic modification based on the hypothesis that repetitive cycles of hypoxia/reoxygenation may increase this cell population. When this system was applied to breast cancer cell lines, a stem-like cell population developed high tumorigenesis [130].

Strikingly, some groups have used normal somatic cells, rather than cancer cells, to generate CSCs. In a recent article by Nagata and co-workers [81] a new method was described. CSCs were originated through the overexpression of OSKM in human somatic fibroblasts. According to this method, induced pluripotent cancer stem-like cells (iCSCs) with self-renewal and pluripotency properties were established through the transduction of exogenous OSKM followed by selection, after embryoid body formation and serial transplantation in immunodeficient mice [81]. Scaffidi and Misteli (2011) generated CSCs from human fibroblasts through the expression of telomerase and oncogenic H-RasV12 mutant and concomitant inhibition of p53 and pRB. The multipotent SSEA-1^+^ cells generated in this process gave rise to hierarchically organized tumours composed of non-tumorigenic SSEA-1^-^ cells [82].

## Clinical applications of CSCs

### Introduction

The currently held theory regarding the abundance of CSCs within a given tumour, although challenged within the studies of Quintana and others, is that is generally very low (<0.1 %) [47, 131, 132]. Given that this population displays high resistance to selected applied therapies, a successful clinical response would come from therapies aiming to omit specifically these rare cells responsible for repopulating the tumour. By targeting the rapidly dividing cancer cells of the bulk tumour, classic chemotherapy is incapable of defeating the low-cycling CSCs. This explains cases where there is an initial visible decrease of primary tumour size as a response to treatment followed by further tumour relapse. A recent study demonstrated that CSCs can be even stimulated by chemo- or radio-therapy, and this was reflected by the higher percentage of CD44^+^CD24^-^ breast CSCs after therapy [133]. It would be perhaps more effective to establish combination of therapies that can target both cancer cells and CSCs [134].

### Development of CSC-specific therapies

As stated above, normal stem cells and CSCs have many properties in common . Therefore, in order for therapies to have a future translational impact, it is important that current research focuses on the identification of CSC-specific properties and of new agents that could be CSC-selective. A study published in 2012 by the group of Bhatia presented a neoplastic hiPSC differentiation system for compound screening of small molecules known to induce cell differentiation [134]. The authors identified thioridazine, a clinically approved drug designed to antagonize dopamine receptors expressed on CSCs, as neoplastic cell- and CSC-specific without affecting normal stem cells, demonstrating at the same time the use of differentiation as a therapeutic strategy. In another line of studies, clinical data were recently presented at the 2015 American Society of Clinical Oncology (ASCO) annual meeting in Chicago regarding the development of two novel compounds, BBI608 and BBI503, orally-administered, which are designed to target CSC pathways in multiple tumour types. BBI608 targets STAT3, leading to inhibition of the critical genes for maintaining cancer stemness. It was also shown to enhance anti-tumour activity when administered with classic chemotherapy in gastric and colorectal cancer. BBI503 inhibits Nanog and other cancer stem cell pathways by targeting kinases, and showed encouraging early signs of anti-cancer activity for patients with advanced colorectal cancer [135]. To date, many CSC-limited therapies based on identified CSC-specific properties have been proposed, many of which hold promise for clinical therapy. Table 2 categorizes these CSC-specific therapies.

**Table 2:** Selected clinical therapeutic agents in current cancer stem cell drug treatment

Target	Name of drug (synonyms)	Mechanism of action	Ref.(s)
Microenviroment/Niche	Anti-CD44 monoclonal Abs	CD44 activation	[249]
	Oblimersen sodium (G3139)	Bcl2 antisense oligonucleotyde	[250]
	AT-101 (gossypol)	Bcl2, Mcl1 inhibitor	[251, 252]
	ABT-263/ABT-737	Pan Bcl2 inhibitor	[253]
	Obatoclax (GX15-070)		[254]
	Sabutoclax (Bl-97C1)		[255]
	Anti-integrin alfa 6 monoclonal Abs	Block integrin alfa 6 binding	[256]
	GLPG0187	Integrin alfa peptide antagonist	[257]
	EMD 121974 (Cilengitide)	Integrin alfa beta peptide antagonist	[258, 259]
	Volociximab	Monoclonal antibody targeting integrin alfa beta	[260]
	ATN-161	Integrin alfa beta peptide antagonist	[261]
	Pepducins	CXCR4 antagonist	[262]
	Plerixafor (AMD3100)		[263]
	Neutralizing ab	CXCR4 blocker	[264]
	Bevacizumab (Avastin)	VEGF-A/VEGFR blocker	[151, 265]
	Cediranib/AZD2171	Tyrosine kinase inhibitor VEGFR2	[152, 266]
	EphA3 monoclonal Abs	EphA3 blockers	Clinical trial phase I by KloBios Pharmaceuticals
	Systemic infusion of enzyme PEGPH20	HA inhibitor	[156]
	Anthracyclines, EGFR inhibitors, cardiac glycosides, histone deacetylases, HSP90 inhibitors, microtubule targeting agents, proteasome inhibitors, topoisomerase I inhibitors	HIF-1 inhibitors	Reviewed in [158]
	DNA vaccines	Legumain, Fra-1, Stat3, FAP, HER-2 (CAFs-TAMs)	Reviewed in [138]
	Monoclonal Ab Sibrotozumab	FAP	Clinical trial phase I [267]; [139]
	PAI-1	uPA/uPAR inhibitors	Reviewed in [137]
	Radioactive labeled Ab, siRNA	Tenascin-C	Reviewed in [137]
	Monoclonal Ab 81C6		Clinical trial phase II [267]; [147]
	NK4, anti-HGF mAbs	HGF/Met	[142, 143]
	5-Aza-2΄-deoxycytidine	DNMT1	[140]
	MMPs inhibitors	MMPs	[141, 268]
Wnt/βcatenin pathway	Anti-Frizzled (Fzd7) monoclonal antibodies (OMP-18R5)	Block formation of active receptor signalling complex; binds 5 Fizzled receptors	Clinical trial phase I by OncoMed; [159, 267]
	Truncated Fizzled 8-Fc fusion protein (OMP-54 F28)	Fzd8-Fc selectively binds Wnt ligands	Clinical trial phase I by OncoMed in collaboration with Bayer Pharma AG
	NSAIDs	COX mechanisms	[269, 270]
	IWP2	o-acetyltransferase inhibitor	[271]
	XAV939	Tankyrase inhibitor	[272]
	PKF115-584	TCF/βcatenin inhibitor	[273]
	CGP049090		
	IWR	Axin stabilizer	[271]
	ICG-001	CREB/βcatenin interaction inhibitor	[274]
Hedgehog pathway	GANT-61	Gli DNA binding inhibitor	[173]
	Arsenic trioxide		[174]
	BMS-833923 (XL139)	SMO inhibitor	[267]
	LDE-225		
	LEQ506		
	PF-04449913		
	TAK-441		
	Cyclopamine and based compounds		[170]
	Vismodegib (GDC-0049)		[275]
mTOR/PI3K/Akt	Perifosine (krx-0401, d-21266)	Akt inhibitor	[276]
	MK-2206		[277]
	GSK690693		[278]
	GSK2141795		[279]
	LY2780301		[280]
	Rapamycin	mTORC1 inhibitor	[281]
	Temisirolimus (CCl-779)		[282]
	Everolimus (SDZ RAD)		[283]
	Ridaforolimus (AP23573, MK-8669)		[284]
	OSI-027	mTORC1/2 inhibitor	[285]
	PP242		[286]
	PP30		
	WAY-600		[179]
	WYE-687		
	WYE-354		
	AZD8055		[287]
	INK128		[288]
	NVP-BKM120	PI3K inhibitor	[289]
	PX-866		[290]
	GDC-0941		[291]
	CAL-101 (GS-1101)		[292]
	SF1126	PI3K & mTOR inhibitor	[293]
	NVP-BEZ235		[294]
	XL765		[295]
	GDC-0980		[296]
	PI-103		[297]
	Metformin	Inhibition of mTOR activation through AMPK	[181]
Notch pathway	Anti-Notch 2 and 3 monoclonal Abs (OMP-595R)	Block cleavage of Notch receptor	Clinical trial phase I by OncoMed
	Anti-Notch 1 and 2 monoclonal Abs		[298]
	Anti-Notch 3 monoclonal Abs		[299]
	Anti-DLL4 (demcizumab)	Block binding of DLLL4 to Notch receptor	Clinical trial phase I by OncoMed
	Anti-DLL4 monoclonal Abs (OMP-21 M18, REGN421)		[160, 267]
	DAPT	Gamma-secretase inhibitor	[300]
	MRK-003		[301]
	LY450139		[302]
Telomerase	Imetelstat/GRN-163 L	Telomerase inhibitor	Clinical trial phase II by Geron Corporation; [216]
	GRN-163	Antagonist of telomerase template	[303]
Drug efflux	PSC-833	ABCB1 inhibitor	[304]
	YHO-13351	ABCG2 inhibitor	[305]
Redox balance and metabolism	Genipin	Suppression of UPC2	[228]
	Phytochemicals	Redox system	[225, 226]
	Indo-3-carbinol (I3C)	Redox system (stimulates BRCA1)	
	Genistein	Redox system (ROS scavenger, inhibitor of NFkB, Akt, PTK)	[227]
CSC immunogenic responses	CD133 peptide, ICT-121	Dendritic cell-based vaccine	Clinical trial phase I by ImmunoCellular Therapeutics
	Peptide vaccine (SL401 and SL701)	Interleukin-3 receptor	Clinical trial phase I and II completed by Stemline Therapeutics
	VS6063	Focal adhesion kinase (inhibitor)	Clinical trial phase I completed by Verastem and Pfeizer
	Ipilimumab	CTLA-4 blockers	Clinical trial phase III by BMS/Medarex; [306]
	TGN1412	CD28 agonist	Clinical trial phase I [267]; [307]
	MDX-1106	PD-1 antagonist	Clinical trial phase I [267]; [308]
	Celebrex, Rofecoxib	COX2 inhibitors	Reviewed in [209]
	CXCR1 blocking Abs or small molecule repertaxin	IL-8/CXCR1 inhibitor	[210]
	TGF-β type II receptor antibody or SMAD4 siRNA	TGF-β/SMAD 4	[211]
Stemness	BBI608	STAT3 inhibitor	Clinical trial phase III [135]
	BBI503	Nanog inhibitor	
miRNA based therapies	let-7 anti-sense oligos	H-RAS and HMGA2	[197]
	miR200c	Bmi-1, ZEB1	[199–201]
	miR-34a	CD44 activation	[202]

### Therapies targeting the CSC microenvironment/niche

The CSC microenvironment is now considered a hot target field in CSC therapy. The idea would be to simply disturb the supportive microenvironment that can feed and support the CSCs population. Niches are the most widely studied microenvironments, defined by a variety of cell types such as cancer associated fibroblasts (CAFs), tumour associated macrophages (TAMs), tumour endothelial cells (TECs), mesenchymal stem cells, immune and endothelial cells lining across the tumour vasculature, cell-cell contacts, extracellular matrix (ECM), and soluble factors such as growth factors and cytokines [136]. Although normal and tumour niches differ in many aspects, manipulation of the conditions of the tumour/metastatic niche may modulate the cancer stem cell frequency, survival and potency. Stromal incompatibility can influence the number of CSCs in human tumours by dramatically affecting the survival of the engrafted human cells within the host, which may be mitigated when humanized mouse models (NSG) are used. Kuperwasser and colleagues (2004), in an effort to reconstruct the human mammary gland within mice, showed that both stromal and epithelial components are necessary for the development of the xenografts in vivo, whereas genetic manipulation of the stromal cells prior to transplantation resulted in the growth of neoplasias. These mouse models, which manage to recapitulate the biological features of patients’ samples, not only provide a unique way to study the steps of cancer pathogenesis in vivo, but also hold promise for individualized cancer therapy, allowing the prediction of patients’ response to clinically tested anticancer regimens [49].

CAFs, TECs, and TAMs are fundamental components of tumour microenvironment, whose role is critical in carcinogenesis and for this reason have attracted a lot of attention as potential targets of tumour microenvironment (reviewed in [137]). CAFs overexpress specific therapy targets such as legumain, an asparaginyl endopeptidase, proto-oncogene Fra-1, transcription factor Stat3, fibroblast activation protein (FAP) as well as HER-2. The development of DNA vaccines directed against some of these targets resulted in the elimination of tumour growth, progression, metastasis and recurrence in mouse tumour models (reviewed in [138]). FAP, a protein that may play a role in the differentiation of fibroblasts to myofibroblasts, can be inhibited via a monoclonal antibody, sibrotuzumab has shown to be promising in early clinical trials [139]. Another approach for CAF-targeted therapy would be the use of drugs that inhibit differentiation of stromal cells into activated CAFs, such as the use of the DNA demethylating agent 5aza-dC (5-aza-2΄-deoxycytidine) to inhibit myofibroblast differentiation from hepatic stellate cells [140]. In addition, certain epithelial cell derived matrix metalloproteinases (MMPs) (i.e. MMP 7, 9, 2 and 3) may play a role in myofibroblast differentiation and in ECM remodelling. MMPs can be targeted in therapy using MMP inhibitors like TIMP-2 [141]. A third approach would be stroma-directed therapies against growth factors, secreted by activated CAFs, which act on epithelial transformation and enhance invasiveness. For example, the hepatocyte growth factor (HGF/Met) pathway has been used as a target in early pre-clinical studies using NK4, a competitive antagonist of Met and anti-HGF monoclonal antibodies [142, 143]. Another CAF-derived factor, the urokinase plasminogen activator (uPA) and its receptor (uPAR) and their mediated signalling have been implicated in tumour cell invasion, survival, and metastasis in a variety of cancers. Inhibitors of uPA and radioactive labelled PAI-1 have shown efficacy in inhibiting tumour growth and invasion in pre-clinical studies of pancreatic and prostate cancers [144, 145] (reviewed in [137]). Tenascin-C, another target of therapy abundantly expressed in CAFs, binds to cell surface receptors and ECM components and has been known to promote colon cancer metastasis in response to TGF-β signalling [146]. Tenascin-C inhibitors and the monoclonal antibody 81C6 have been shown to be effective in early clinical trials in astrocytomas and Non-Hodgkin’s lymphoma [137] and in brain tumours [147], respectively. The CAFs-related connective tissue growth factor (CTGF) holds a therapeutic promise. Aikawa et al. (2006) developed a fully humanized CTGF-specific monoclonal antibody, FG-3019, as a novel therapeutic approach for pancreatic ductal adenocarcinoma. FG-3019 decreased the volume of intra-pancreatic tumour and attenuated its metastatic potential [148].

The prevention of the growing vascularisation in the tumour can be used as a CSC-target therapy. Vascular endothelial growth factor (VEGF), a molecule that directly supports the development of local vasculature, is the target of bevacizumab [149]. When glioma and medulloblastoma were treated with this inhibitor, a depletion of CSCs was observed, demonstrating that VEGF is a relevant target for CSCs [150]. Promising results of anti-angiogenesis therapy have been reported in clinical trials for bevacizumab [151] or cediranib (AZD2171, a VEGFR inhibitor) [152]. Interactions with the stromal microenvironment can alter the survival pathways that are activated in CSCs. One example might be the culture of pro-survival proteins, like Bcl-2 and Bcl-X_L_ with CD34^+^ leukaemia CSCs, thus diminishing apoptosis after chemotherapy [153]. In addition to VEGF inhibitors, a high number of Bcl-2 family inhibitors are currently in preclinical and clinical drug development [154].

An anti-EphA3 antibody, currently in phase I trial, offers another potential therapy acting on the microenvironment. EphA3 was found to be expressed in the preB leukaemia cell line and in a subset of samples from patients with leukaemia [155]. Although no direct link was found between EphA3 expression and LSCs, studies have shown that anti-EphA3 may act against CSCs, since its incubation with preB leukaemia cells leads to the loss of the CSC’s in vitro ability to form colonies. In a study by Provenzano et al. (2012), when an enzyme (PEGPH20) targeting hyaluronic acid (HA) is systemically delivered with standard chemotherapy it can permanently remodel the tumour microenvironment and achieve better anti-tumour responses, resulting in a near doubling of the overall survival [156]. A detailed list of drugs targeting various components of the niche is listed in Table 2.

The hypoxic niche is another area of significant interest. The slow proliferation or quiescence that normal stem cells exhibit under low O_2_ conditions resembles that of cancer stem cells’ genetic-epigenetic adaptation to the hypoxic conditions in the centre of a poorly vascularised and oxygenated tumour. The brain CSCs, which reside in low oxygenated perivascular niches inside the bulk tumour, were shown to be specifically increased by hypoxia [149]. A hypoxic niche for glioma cells was described by Jeremy Rich’s laboratory showing that hypoxic conditions resulted in reprogramming of CD133^-^ cells to CD133^+^ with enhanced CSC-phenotype [157]. A major mechanism mediating the adaptation to hypoxia is the activity of transcription factor HIF-1. Existing pharmacological data demonstrate the anti-cancer effects of HIF-1 inhibitors and drug-efflux pump inhibitors, as monotherapy and/or as sensitizers to chemotherapy in mouse models of human cancer [158].

### Therapies targeting central signalling pathways of CSC survival

Other drug candidates may target the Wnt, mTOR/PI3K/Akt, or Notch pathways, involved in both CSC and adult stem cell signalling [159, 160]. The Wnt pathway plays a central role in a whole spectrum of different developmental processes, including cell proliferation and migration, being crucial in cell fate and tissue patterning [161, 162]. It is already known that the Wnt pathway is involved in the self-renewal of both normal and cancer stem cells. Indeed, aberrant Wnt signalling was observed in the CSC population from many tumour types [161]. The high number of Wnt isoforms and the complicated mechanism of the Wnt pathway may explain why most of its inhibitors are still in the preclinical stage. Extirpation of CSCs by Wnt-selective drugs without depleting the normal haematopoietic stem cells (HSCs) in G_1_ phase, cells depending on the Wnt pathway, might be limited. Instead, the non-steroid anti-inflammatory drugs (NSAIDs), indomethacin or sulindrac, have been proved to act on the Wnt/βcatenin pathway [163, 164], as have the second generation cyclooxygenase 2 (COX2) inhibitors like celecoxib [165]. Lately, a downregulation of the Wnt inhibitory factor 1 (WIF1) as a widespread event in salivary gland carcinoma ex-pleomorphic adenoma (CaExPA), has been described [163, 166]. WIF1 downregulation occurs in the CaExPA precursor lesion pleomorphic adenoma (PA) and indicates a higher risk of progression from benign to malignant tumour [166]. Most importantly, WIF1 significantly diminished the number of salivary gland CSCs and the anchorage-independent cell growth. Consistent with this observation, WIF1 caused a reduction of the expression of pluripotency and stemness markers as well as the adult stem cell self-renewal and multi-lineage differentiation markers WNT3A, TCF4, c-Kit, and Myb [166]. Detailed names and corresponding references of candidate drugs targeting the Wnt pathway are listed in Table 2.

The Hedgehog (HH) pathway is involved in the maintenance and regeneration of adult tissues, through the regulation of adult stem cells, as well as in cancer development through the regulation of CSCs [167]. Currently three mammalian HH ligands have been identified, of which Sonic HH (SHH) is the best described and studied. In the absence of HH ligand, its receptor called Patched (Ptch) represses Smoothened (SMO). Binding of HH removes this repression, allowing SMO to activate Gli family of transcription factors and other targets [168].

Although the HH pathway can be activated at several different points, most drugs target the SMO transmembrane protein. The first inhibitor to be identified was cyclopamine, which directly binds to SMO to efficiently block the HH pathway and decrease CSCs number in preclinical models [169, 170]. Unfortunately, the use of cyclopamine is limited due to its low oral bioavailability and pharmacokinetics [171]. The first clinically approved antagonist of HH is vismodegib (GDC-0049). Several more small molecules are currently being clinically tested like PF-04449913 or TAK-441 among others (Table 2). The HH pathway can also be a target by upstream ligands or by downstream effectors. Antibodies targeting SHH have been shown to inhibit the clonogenic potential of CSCs [172]. Inhibitors of Gli transcription factors, such as the Gli DNA binding inhibitor GANT-61 [173] and arsenic trioxide [174], may significantly reduce self-renewal in tumours with mutations that are located downstream of SMO, or which simply acquire resistance to SMO-targeted molecules.

The mTOR/PI3K/Akt pathway is another known CSC therapeutic target as it has been shown to be especially involved in the generation and survival of leukaemia [175]. Many different mTOR inhibitors were already developed and tested, including rapamycin itself and rapamycin-related drugs (e.g. CCI-779 and RAD001). Although these molecules were shown to inhibit CSC self-renewal [176], clinical use had limited success [177, 178]. Meanwhile, novel ATP-competitive inhibitors that can target both mTORC1 and mTORC2 complexes have been tested [179]. Alternatives are a combination of different mTOR/PI3K/Akt inhibitors that have been proven to be effective in CSCs and some of them are currently in phase 1 of clinical trials. Gemcitabine, a nucleoside analogue used in chemotherapy, when combined with rapamycin significantly reduced the percentage of pancreatic CSCs in vitro obtained from primary patient samples [180]. An alternative therapeutic candidate of mTOR inhibitors is metformin, a well-known drug for the treatment of type 2 diabetes. Metformin blocks mTOR through AMPK activation, important in protein synthesis, cell cycle progression, and cell survival [181]. A combination of metformin with chemotherapy displays prolongation of survival of breast, lung and prostate cancers [182, 183]. Shank and colleagues (2012) observed an inhibition of ovarian CSCs growth in vitro caused by metformin, dissimilar to what was observed in vivo. However, when combined with cisplatin, a chemotherapeutic drug tested in different types of cancer, a restriction of the tumour growth in vivo was observed [184]. Detailed names and corresponding references of candidate drugs targeting the mTOR/PI3K/Akt pathway are listed in Table 2.

Notch signalling, another stem cell-supporting pathway, is activated in many human cancer types and its deregulation is related with this disease. The Notch pathway has been reported to play an important role in the regulation of asymmetric cell fate decisions in human mammary stem cells [185]. Abnormal activation of the Notch pathway has been observed in CSCs from some tumours, mostly related to self-renewal maintenance and resistance to therapy observed in these cells [186]. Therefore, inhibition of the Notch signalling pathway could be a therapeutic target for the elimination of the CSC subpopulation [187]. Preclinical analysis in leukaemia, medulloblastoma and breast cancer with the gamma-secretase inhibitors DAPT [79, 188], GSI-18 [189, 190], and MRK-003 [191], showed selective targeting of CSCs and reduced tumour sizes (Table 2). Moreover, the Notch pathway could be a direct target of miRNAs closely related to invasion and acquisition of stem cell like properties by tumour cells [192]. More drugs targeting different components of the Notch pathway are listed in Table 2.

### Therapies targeting CSC-miRNAs

miRNAs are relatively small noncoding RNA molecules that serve as transcriptional and post-transcriptional repressors through the binding to their mRNA targets [193]. Taking into account that different types of cancer have specific profiles of miRNA expression reminiscent of their stem cell of origin [194], the application of miRNA-based tools in cancer therapy has lately attracted a lot of interest [193]. miRNAs appear attractive therapeutic targets in cancer as they may affect the expression of multiple genes in different developmental contexts, including regulation of critical stem cell features as well as metastasis, EMT, and cell division. For these reasons, many miRNAs have been successfully targeted in vivo in mouse models with the use of specific inhibitors called antagomiRs [195] or have been proposed as targets in exosome biology, by either targeting the release of miRNAs from exosomes, or by using these “bioactive vesicles” for therapeutic delivery (reviewed in [196]).

In a study on breast cancer cell lines and human primary cancer cells let-7a miRNA, negative regulator of stemness, was found significantly decreased in breast CSCs and increased with differentiation [197]. Re-expression of let-7 miRNA in CSCs using a lentivirus construct causes a reduction of the CSC proliferation and mammosphere formation. Moreover it also negatively influenced the ability of CSCs to form tumours and metastasis in mice [197]. In another study of 2009, 37 miRNAs were described as being up- or down-regulated in CD44^+^CD24^-^ breast CSCs when compared to non-tumorigenic cells [198]. Shimono and coworkers focused mostly on BMI1 since it is known to regulate normal and CSC activity [199, 200]. Exogenous miR-200c was found to promote reduction of BMI1 protein levels and embryonal carcinoma cells growth in vitro and in vivo in NOD/SCID mice [199, 200]. In normal mouse breast stem cells expression of miR-200c repressed outgrowth formation in the mammary fat pad assay [200], while miR-200c expression in human breast CSCs blocked tumour initiation in NOD/SCID mice [197, 201]. miR-34a-based therapies are emerging as novel modes of therapeutic interventions. Systemic delivery of miR-34a, which is known to target CD44 and is usually repressed in prostate cancer, inhibited metastasis of prostate cancer cells and prolonged survival of mice [202].

### Therapies targeting CSC-immunogenic responses

Immune tolerance is an important property of a growing tumour. Complexed immunogenic reactions taking place within the tumour microenvironment are responsible not only for tumour’s survival against the immune system but also for the enhancement of its stemness and migration properties. In a study of Giannoni et al. (2010), IL-6 was shown to activate the CAFs, which in turn induced EMT and stemness of prostate cancer cells [203]. In support of this, Iliopoulos et al. (2011) identified IL-6 as the mediator responsible for conversion of non-stem cancer cells into CSCs in breast and prostate cancer [204]. Therapy-induced changes in the tumour stroma play a critical role in determining phenotypic plasticity and may be responsible for pro-malignant phenotype acquired by the surrounding CAFs [205]. Such events give rise to a pro-inflammatory microenvironment characterized by increased expression of TNFα, pro-inflammatory cytokines, and INFγ, which finally induce STAT3/NFkB signaling responsible to promote self-renewal in ESCs [206] but also to mediate resistance to therapy [207]. In another study, it was reported that mesenchymal cells in breast cancer support CSCs through a signalling loop dependent on IL-6 and CXCL7 [208].

For these reasons, many pharmaceutical companies today aim in the targeting of CSC subpopulation based on selected immunogenic responses. Most promising from current clinical trials are candidates that are related with CSC-associated proteins used as antigens to elicit an immune response against CSCs (Table 2). Other drugs, such PD-1 and COX2 inhibitors and CD28 agonists, have been proposed as promising anti-cancer therapies Some of them, like celecoxib and rofecoxib, have both been tested in phase II clinical trials in combination with chemotherapeutic agents in patients with NSCLC, and pancreatic, breast, and colorectal cancers and, for the most part, have shown additional clinical benefit beyond that observed with chemotherapy alone (reviewed in [209]). In another study, Ginestier et al. (2010) developed a strategy to deplete selectively breast CSCs in preclinical models through blockade of the IL-8 receptor CXCR1 using either a CXCR1-specific blocking antibody or repertaxin, a small-molecule CXCR1 inhibitor [210]. Blockade of TGF-β/SMAD signaling pathway by the TGF-β type II receptor antibody or SMAD4 siRNA repressed enrichment of CSCs in the triple negative breast cancer cell model [211]. Additional candidate drugs targeting immunogenic responses of CSCs have been proposed and some of them have reached the clinical phase (Table 2).

### Therapies targeting telomeres and telomerases

Another example of CSC-specific targets is telomeres and telomerases. Regions of repetitive nucleotide sequences called telomeres protect the end of the chromosomes from being recognized as double-strand breaks by the DNA repair system [212]. Classic stem cells display constitutive activity of telomerase, an enzyme that adds DNA sequence repeats to the telomere regions, and its presence in cancer cells can be a reflection of an origin of those cancers from stem/progenitor cells and the imposition of maturation arrest [213]. Compared to normal stem cells, cancer stem cells express higher levels of telomerase [214]. Based on that, telomerase could be a relevant target in CSCs without destroying healthy stem cells. The most common approach to target telomerase activity is immunotherapy and the use of oligonucleotide-based hTERT inhibitors [215], both listed in Table 2. Widely studied telomerase inhibitors are GRN163 and a more potent lipid attached derivative, GRN163L (imetelstat) [216]. The telomerase specific drugs lead to the reduction in the self-renewal capacity of treated CSCs as assessed by in vitro sphere formation [217] (Table 2). One telomerase related drug imetelstat, was shown to block replication of glioblastoma CSCs and to decrease tumour growth [218]. Furthermore, telomerase inhibitors are expected to target also the bulk of the tumour. Treatment of glioma, neuroblastoma, lung and prostate CSCs with imetelstat induces reduction in the self-renewal capacity of these cells as assessed by the in vitro sphere formation. Another finding was that this drug displays effective properties against xenograft tumours and reduced the number of CSCs remaining in treated tumours [218].

### Therapies targeting CSC redox balance and metabolism

Another feature that characterizes CSCs and has been under investigation for clinical targeting is the redox balance. Oxidative stress refers to a condition of the cell characterized by an excess of reactive oxygen species (ROS) [28]. ROS, such as superoxide, hydrogen peroxide, and peroxynitrite, are physiological byproducts of mammalian metabolism, and in excess may cause cell damage by oxidation and nitration of macromolecules, such as DNA, RNA, proteins, and lipids [219]. A cell generally undergoes apoptosis when ROS levels are high or goes into senescence at sublethal levels [220]. In turn, ROS may play a role in cell proliferation, differentiation, and apoptosis through the control of signalling pathways [221].

The subsistence of the redox status is important for normal stem cells to avoid cellular differentiation, to maintain long-term self-renewal and to decrease the accumulation of DNA damage [28, 222]. This is indicated by the general low levels of ROS in normal stem cells compared to their differentiated progeny, as shown in normal HSCs and progenitors, central nervous system stem cells and normal breast stem cells [223, 224]. The resistance to oxidative stress is regulated by the ROS scavenging system that is upregulated in these cells. Cellular redox status is maintained by several intracellular redox-regulating molecules, including thioredoxins (TRX), and glutathione (GSH)/glutaredoxin (GRX). Molecules or enzymes that can destabilize the redox status of cancer cells will be beneficial for actually killing them. In fact, the combination of chemotherapy with cellular redox system modifiers has shown promising results in clinical trials. For example targeting redox system with phytochemicals, natural substances derived from vegetables, fruits and spices, is another method described as being capable of intervening in carcinogenesis, which has already reached clinical trials both in combination with, and without, conventional chemotherapy [223]. In another study, a phytochemical from cruciferous vegetables, the indole-3-carbinol (I3C), was associated with a reduced risk of several tumour types, such as breast and prostate cancers. In the acidic environment of the stomach, I3C undergoes hydrolysis to a number of products, including a dimeric product, 3,3'-diindolylmethane (DIM). Both of these phytochemicals stimulate BRCA1 in breast and prostate cancer cells and have been shown to protect cells against oxidative stress mediated by H_2_O_2_ and γ-radiation [225, 226]. Another natural substance, genistein (4,5,7-trihydroxyisoflavone), which was identified as the predominant isoflavone in soybean enriched foods, has been shown to inhibit prostate carcinogenesis in animal models. Genistein has antioxidant effects and protects cells against ROS by scavenging free radicals, inhibiting the expression of stress-response related genes [226]. Furthermore, genistein is a powerful inhibitor of NFkB, Akt and PTK signalling pathways, all of which are important for cell survival [227].

Moreover, since mitochondria are the main source of ROS, its limitation can be achieved by switching metabolic pathways from oxidative phosphorylation to aerobic glycolysis, a phenomenon known as the “Warburg effect”, which is characteristic of normal and CSCs. The mitochondrial uncoupling enzymes UCP2 are considered responsible for this metabolic change and this is the main reason why they are in the focus of drug development. Suppression of UCP2 by genipin, a plant derived small molecule, was shown to suppress tumorigenic properties of breast cancer cells, mediated by a decrease of ROS and downregulation of UCP2 [228].

### Biomarker discovery and cancer clinical diagnostics

In order to reduce the rate of adverse clinical outcome among patients, it is very important to detect cancer in early stages as well as to identify which patients are more likely to benefit from available therapies. In this way, identification of biomarkers and their application in the screening of cancer are of enormous importance as they would allow proper detection and prognosis of cancer [229]. Biomarkers, molecules found in tissues or fluids within the body of an individual and related with a disease [230], can be either DNA, RNA, miRNA, epigenetic modifications or protein expression [51]. Biomarkers may be used to detect cancer, risk assessment, distinction between benign and malignant forms of cancer, disease status, response to therapy, tumour recurrence and many other applications [231]. Kim et al. (2013) developed an in vivo model of pancreatic cancer at an early stage of the disease that can be advantageous in the identification of biomarkers related with this phase of the disease and applicable to disease monitoring after therapy [69]. Three month old teratomas, generated from the injection of reprogrammed human pancreatic ductal adenocarcinoma (PDAC) cells into immunodeficient mice, exhibited pancreatic intraepithelial neoplasia (PanIN) structures and marker expression related to the early stage of the disease. When teratomas were observed 6-9 months after injection, a progression to invasive stage of human pancreatic cancer was detected. Indeed, proteins collected from the serum-free media and analysed by nanoLC/MS/MS enabled the identification of secreted or released proteins associated with the activation of HNF4α specific to the late PanIN stage [69]. This discovery can be used as a tool in the diagnostics and disease monitoring after therapy of pancreatic cancer.

Recent technological advances have allowed the possibility to analyse the characteristics of circulating exosomes and microvesicles secreted by CSCs for cancer detection and monitoring. Exosomes, small endocytic membrane-derived vesicles important for cell homeostasis and cell-to-cell communication present in most bodily fluids, can harbour a variety of proteins, nucleic acids, and lipids. Exosomes can merge with and release their contents in recipient cells and can exhibit a broad range of functions, can mediate adaptive immune responses to pathogens and tumours, promote tumorigenesis, angiogenesis, and metastasis, and can determine response to therapy by transferring oncogenes and onco-miRNAs between cancer cells or between the tumour and its stromal environment. Exosomes are very stable and due to their phospholipid bilayer protect their contents from degradation by nucleases and proteases. Consequently, biomarkers at a relatively low expression are much easier to be detected through isolating exosomes. For instance, some biomarkers such as PCA3 and TMPRSS2 are mRNAs not easily detected in body fluids, but appear in exosomes in prostate cancer [232].

CD63^+^ exosomes were significantly increased in plasma of melanoma patients compared to healthy controls [233], while in a comparative analysis, CD63 was found enriched in exosomes derived from malignant cancer cells compared to those derived from non-cancer cells [234]. In another study, glioblastoma-specific epidermal growth factor receptor vIII (EGFRvIII) was found in serum exosomes from a significant proportion of patients, whereas EGFR, EGFRvIII, and TGF-β were found in serum exosomes from patients with brain tumours, suggesting that they all might provide diagnostic information for glioblastomas and brain tumours respectively [235, 236]. Since 2009, when it was reported that miRNAs, previously demonstrated as diagnostic markers for ovarian cancer, were found at similar levels in biopsy specimens of ovarian cancer and serum exosomes isolated from the same ovarian cancer patients, many cases of exosomal miRNAs have been proposed for cancer diagnostics. Brase and coworkers showed that serum levels of miR-141 and miR-375 correlate with tumour progression in prostate cancer [237]. Moreover, the exosomal miR-21 level was found elevated in serum from patients with esophageal squamous cell cancer (ESCC) versus serum from patients who have benign tumors without systemic inflammation [238]. Interestingly, Takeshita and colleagues reported that serum-derived exosomal miRNA-1246 was a good biomarker that significantly correlated with the metastasis stage and was found to be a strong independent risk factor for poor survival [239]. In a study of stage I involving PDAC patients, higher levels of exosome-resident macrophage migration inhibitory factor (MIF) were found to predict an increased risk of the eventual development of liver metastasis [240].

In contrast to exosomes, which originate from endosomes, microvesicles are fragments that derive directly from the plasma membrane, with a size range from 100 nm to 1 μm. The group of Camussi demonstrated recently that microvesicles derived from CSCs can induce angiogenesis and lung metastasis in vivo [241]. The group of researchers isolated CD105^+^ cells from human renal carcinoma and showed they possess CSC characteristics like expression of stem cell markers, formation of spheres in vitro, and ability to form tumours in vivo. The CSC-derived microvesicles were CD105^+^ and contained mRNA and miRNA. The mRNA (e.g. VEGF and FGF2) was shown to be involved in the stimulation of angiogenesis as confirmed by the formation of capillaries in vivo after injection of microvesicle-treated HUVEC. When renal tumour cells were injected together with CSC-derived microvesicles into mice, a higher number of metastasis was observed compared to CD105^-^ cells derived-microvesicle [241].

As already mentioned, CAFs do not just surround the growing tumour passively, but are actively participating in the establishment of a metastasis-promoting communication, influencing at the same time innate and adaptive immune responses. For these reasons, CAFs and their products are now considered potential prognostic biomarkers and candidate targets for novel therapeutic strategies (reviewed in [242]). In CRC stage II and III, α-smooth muscle actin (a-SMA)-expressing CAFs were proposed as useful indicators of poor prognosis [243], whereas the combination of calumenin with cadherin 11 expressed by CAFs displayed a significant association with disease-free survival and overall survival [244]. Moreover, in patients with advanced colorectal adenocarcinomas, increased stromal FAP was considered an adverse prognostic marker [245]. A significant correlation was observed between stromal FAP-a and stromal cell-derived factor-1 (SDF-1) mRNA levels, primarily expressed by CAFs, after pre-operative chemo/radiotherapy in rectal cancer patients. FAP-a and SDF-1 gene expression patterns significantly correlated with distant recurrence and poor probability of recurrence-free and overall survival [246]. In another study, multiplex bead immunoassays and an enzyme-linked immunosorbent assays were used to characterize CAFs-signatures from 52 kinds of CAFs in 68 GC patients who were treated with fluoropyrimidine and platinum combination chemotherapy. Serum CAF profiling was able to differentiate GC patients in groups, and more importantly, distinguishing a high 11-CAF signature could identify GC patients with a poor prognosis when treated with standard chemotherapy who need urgent new treatment strategies [247]. Accordingly, Tchou and colleagues observed different gene expression profiles (particularly in pathways associated with cytoskeleton, integrin signalling and metastasis) among early passage primary CAFs derived from three main subtypes (ER^+^, triple negative and Her2^+^) of human breast cancer samples, suggesting that CAFs gene expression profile might be a useful marker in breast cancer prognosis [248].

## Conclusions and further directions

More and more evidence is currently supporting the CSCs theory. It is known that CSCs play a crucial role in cancer initiation and primary tumour sustainment. These cells can also facilitate the metastasis to distant organs from the primary site. Based on these CSCs properties, it is obvious that they are excellent therapeutic targets, assuming that elimination of the CSC population will block the metastasis and eliminate the possibility of tumour regeneration. Since not only CSCs but also stem cells are able to protect themselves from cellular insult it would be important to probe whether blocking either normal stem cells properties or related pathways can induce CSC sensitivity to selected therapies. Many prevalent treatments were founded based on stem cell pathways: Notch, Hedgehog, or Wnt, among others. Moreover, not only the pathways themselves, and surely not only one single pathway, are important in order to enrich CSCs, but many intra-microenvironmental connections, and relationships between microenvironment and the tumour, play a crucial role in enriching this subpopulation. Also a fundamental step before directly targeting the CSC subpopulation would be characterization of the complex and functional markers. Development of clinical trials related to biomarker strategies would be essential to identify proper endpoints to clinically assess therapies targeting a minority, in most type of cancers, of tumour cells. As already described, the CSC population is heterogeneous. New data obtained with CSC in vitro models provided a broadened knowledge about the nature of these cells. On the top of that, CSCs could give us clues about the identification of stem cell pathways activated during cancer progression, which could guide further therapeutic steps and the precise design of preclinical and clinical trials.

Although further studies are needed to explore the relevance of CSCs in vitro models, it is certainly true that the data based on them have a potential utility for clinical applications. Such prospective therapies also have significant implications for pharmaceutical companies that are seeking to successfully develop a drug in late clinical phases.

## Abbreviations

5-azaC: azacitidine
ABC: ATP-binding cassette
ALDH: Aldehyde dehydrogenase
AML: Acute myelogenous leukaemia
APL: Acute promyelocytic leukaemia
a-SMA: α-smooth muscle actin
CaExPA: Carcinoma ex-pleomorphic adenoma
CAF: Cancer associated fibroblast
COX2: Cyclooxygenase 2
CSC: Cancer stem cell
CTGF: Connective tissue growth factor
EC: Human embryonal carcinoma
ECM: Extracellular matrix
EGF: Epidermal growth factor
EGFRvIII: Epidermal growth factor receptor vIII
EMT: Epithelial-mesenchymal transition
ESC: Embryonic stem cell
ESCC: Esophageal squamous cell cancer
FAP: Fibroblast activation protein
FBS: Foetal bovine serum
GJIC: Gap junctional intercellular communication
GRX: Glutaredoxin
GSH: Glutathione
HA: Hylouronic acid
HDAC: Histone deacetylase
HGF/Met: Hepatocyte growth factor
HH: Hedgehog pathway
HIF: Hypoxia-inducible factor
HSC: Haematopoietic stem cell
I3C: Indole-3-carbinol
iCSC: Induced pluripotent cancer stem-like cell
iPC: Induced pluripotent cancer cell
iPCSC: Induced pluripotent cancer stem cell
iPSC: Induced pluripotent stem cell
LSC: Leukaemia initiating stem cell
MIF: Migration inhibitory factor
miRNA: microRNA
MMP: Matrix metalloproteinase
NOD/SCID: Non-obese diabetic severe combined immunodeficient
NSAID: Non-steroid anti-inflammatory drug
NSCLC: Non-small cell lung cancer
NSG: Non-obese diabetic scid gamma mice
NT: Nuclear transfer
OSKM: Oct4, Sox2, Klf4, and c-Myc
PA: Pleomorphic adenoma
PanIN: Pancreatic intraepithelial neoplasia
PDAC: Pancreatic ductal adenocarcinoma
PPARg: Peroxisome proliferator activated receptor gamma
ROS: Reactive oxygen species
SAHA: Suberoylanilide hydroxamic acid
SCID: Severe combined immunodeficient
SDF-1: Stromal cell-derived factor-1
SHH: Sonic Hedgehog pathway
SP: Side population
TAM: Tumour associated macrophage
TEC: Tumour endothelial cell
TRX: Thioredoxin
TSA: Trichostatin A
uPA: urokinase plasminogen activator
uPAR: urokinase plasminogen activator receptor
VA: Valproic acid
VEGF: Vascular endothelial growth factor
WIF1: Wnt inhibitory factor 1
